# Elucidating T cell dynamics and molecular mechanisms in syngeneic and allogeneic islet transplantation through single-cell RNA sequencing

**DOI:** 10.3389/fimmu.2024.1429205

**Published:** 2024-07-19

**Authors:** Hairong Zhou, Zuhui Pu, Ying Lu, Peilin Zheng, Huizhen Yu, Lisha Mou

**Affiliations:** ^1^ Department of Cardiology in South Branch, Shengli Clinical Medical College of Fujian Medical University, Fuzhou, Fujian, China; ^2^ Department of General Medicine, People’s Hospital of Longhua, Shenzhen, Guangdong, China; ^3^ Imaging Department, Institute of Translational Medicine, Health Science Center, The First Affiliated Hospital of Shenzhen University, Shenzhen Second People’s Hospital, Shenzhen, Guangdong, China; ^4^ MetaLife Lab, Shenzhen Institute of Translational Medicine, Shenzhen, Guangdong, China; ^5^ Department of Cardiology in South Branch, Fujian Provincial Hospital, Fuzhou, Fujian, China; ^6^ Department of Geriatric Medicine, Fujian Provincial Hospital, Fuzhou, Fujian, China

**Keywords:** diabetes, islet transplantation, allotransplantation, single-cell RNA sequencing, T-cell, immunomodulation, transplant rejection, immune tolerance

## Abstract

Islet transplantation is a promising therapy for diabetes treatment. However, the molecular underpinnings governing the immune response, particularly T-cell dynamics in syngeneic and allogeneic transplant settings, remain poorly understood. Understanding these T cell dynamics is crucial for enhancing graft acceptance and managing diabetes treatment more effectively. This study aimed to elucidate the molecular mechanisms, gene expression differences, biological pathway alterations, and intercellular communication patterns among T-cell subpopulations after syngeneic and allogeneic islet transplantation. Using single-cell RNA sequencing, we analyzed cellular heterogeneity and gene expression profiles using the Seurat package for quality control and dimensionality reduction through t-SNE. Differentially expressed genes (DEGs) were analyzed among different T cell subtypes. GSEA was conducted utilizing the HALLMARK gene sets from MSigDB, while CellChat was used to infer and visualize cell-cell communication networks. Our findings revealed genetic variations within T-cell subpopulations between syngeneic and allogeneic islet transplants. We identified significant DEGs across these conditions, highlighting molecular discrepancies that may underpin rejection or other immune responses. GSEA indicated activation of the interferon-alpha response in memory T cells and suppression in CD4+ helper and γδ T cells, whereas TNFα signaling via NFκB was particularly active in regulatory T cells, γδ T cells, proliferating T cells, and activated CD8+ T cells. CellChat analysis revealed complex communication patterns within T-cell subsets, notably between proliferating T cells and activated CD8+ T cells. In conclusion, our study provides a comprehensive molecular landscape of T-cell diversity in islet transplantation. The insights into specific gene upregulation in xenotransplants suggest potential targets for improving graft tolerance. The differential pathway activation across T-cell subsets underscores their distinct roles in immune responses posttransplantation.

## 1 Introduction

Islet transplantation has become a promising therapy for certain endocrine disorders, particularly type 1 diabetes mellitus (T1DM), which affects approximately 1.6 million Americans, and the incidence of this disease continues to increase globally (1–3). Despite advances in insulin therapy and continuous glucose monitoring, achieving optimal glycemic control remains a challenge for many patients, leading to long-term complications and increased mortality (4, 5). The limitations of current treatments underscore the urgent need for alternative approaches, such as islet transplantation, to restore endogenous insulin production and achieve tighter glycemic control. However, the success of such transplantations is often limited by immune rejection and the scarcity of donor islets. Recent advancements have explored the feasibility of using personalized endoderm stem cell-derived islets, which may provide a renewable source of islet tissues tailored to individual patient needs, potentially overcoming the limitations of donor availability and improving the compatibility and longevity of grafts (6). Moreover, Encapsulation techniques, which protect transplanted islets from the immune system using biomaterials, offer a potential solution to enhance graft survival and function (7). Targeted local drug delivery systems have also been developed to modulate immune responses directly at the transplantation site, thereby improving transplant outcomes by addressing non-specific, alloantigen-specific, and autoimmune rejection pathways (8).

Allogeneic islet transplantation has demonstrated efficacy in restoring insulin independence in T1DM patients; however, donor scarcity and the necessity for chronic immunosuppression limit its widespread application (8–10). Moreover, allogeneic islet transplantation is also accompanied by significant immunological challenges, primarily due to robust T-cell-mediated rejection (11). The critical role of T-cell dynamics in islet transplantation is underscored by their central involvement in immune tolerance and rejection processes. Understanding the molecular mechanisms governing T-cell responses is crucial for improving graft survival and function. A detailed study of these dynamics can provide insights into more effective immunosuppressive therapies and long-term graft survival, addressing both the immediate and prolonged challenges that impact the success of allogeneic transplants.

The critical role of T-cell dynamics in islet transplantation is underscored by their central involvement in immune tolerance and rejection processes. Detailed study of these dynamics can provide insights into more effective immunosuppressive therapies and long-term graft survival.

The heterogeneity of T-cell subpopulations and their distinct roles in transplantation immunobiology has been studied. For instance, regulatory T cells have been shown to promote graft tolerance (12), while effector T cells contribute to graft rejection (11). The phenotypic characterization of T-cell subpopulations in the context of islet transplantation has revealed potential targets for immunomodulatory therapies, indicating the potential of these cell types for improving transplantation outcomes (13).

In this study, we utilized cutting-edge single-cell RNA sequencing (scRNA-seq) technology (14) to dissect the molecular mechanisms, gene expression profiles, biological pathway alterations, and intercellular communication patterns among T-cell subgroups in both allogeneic and syngeneic islet transplantation models. This approach provided a high-resolution view of the cellular heterogeneity and dynamic changes within the T-cell community, essential for pinpointing the critical factors influencing transplantation outcomes. Our comprehensive analysis using scRNA-seq, along with Gene Set Enrichment Analysis (GSEA) and CellChat, enabled us to uncover significant genetic variations and differences in gene expression between transplantation conditions, revealing the activation or suppression of specific biological processes and signaling pathways within different T-cell subpopulations. These findings offer new insights into the complex communication patterns among T-cell subgroups and with other cell types, highlighting differences in signaling activities between allogeneic and syngeneic transplants that could be pivotal for developing targeted therapeutic strategies.

## 2 Materials and methods

### 2.1 Single-cell data analysis of islet grafts

The analysis of single-cell RNA sequencing (scRNA-seq) data from syngeneic and allogeneic islet grafts was meticulously conducted to understand the cellular heterogeneity and underlying molecular mechanisms that differentiate these two transplant types by Seurat (version 4.1.0) (15). We obtained the scRNA-seq datasets from our previous study (GSE198865) (16). These datasets included samples from both syngeneic (genetically identical donor and recipient) and allogeneic (genetically different donor and recipient) islet transplantation. Cells were filtered using the Seurat package according to the following criteria (1): Cells with fewer than 200 detected genes or more than 4,500 genes were removed to exclude empty droplets or multiplets, respectively. (2) Cells with a mitochondrial gene content exceeding 15% were also excluded to avoid cells undergoing apoptosis or those with damaged membranes. (3) Genes not detected in at least 3 cells were removed to focus the analysis on biologically relevant transcripts. Following rigorous computational quality filtering, we successfully obtained the transcriptomes of 19,640 single cells, comprising 11,870 cells derived from allografts and 7,770 cells originating from syngeneic grafts. PCA was performed to reduce the dimensionality of the dataset and to highlight the genetic variances that differentiate cells. We utilized the “RunHarmony” function (17) within Seurat to correct for potential batch effects across different samples, ensuring that subsequent analyses were not confounded by technical variations.

The t-SNE was used to visualize the data (18). Based on known marker genes, cells were annotated to identify specific cell types, such as lymphocytes, endothelial cells, islet cells, mesenchymal cells, and myeloid cells. Following the initial preprocessing and clustering, further analysis was conducted to delineate the cellular subtypes of T cells (including 6471 cells) and understand their functional roles within the grafts.

The analysis revealed distinct T cell populations in the transcriptome data. Among these, the CD4+ Tconv (Conventional CD4+ T Cells) were characterized by the presence of Cd4 and Tnfsf8 markers. The Activated CD8+ T Cells were identified using Cd8a and Klrc1 markers. Regulatory T Cells, also known as Tregs, were distinguished by Il2ra and Foxp3 markers. The Dividing T Cells were recognized by Stmn1 and Top2a markers, indicating their proliferative state. Memory T Cells were characterized by Sell and Ccr7 markers. Finally, Gamma Delta T Cells were identified based on the presence of Blk, Cd163l1, and Rorc markers.

### 2.2 Analysis of differentially expressed genes

To explore the molecular differences between T-cell subsets derived from syngeneic and allogeneic islet grafts, we used a rigorous approach to identify differentially expressed genes (DEGs). Initially, DEGs were screened using the Wilcoxon rank-sum test. This nonparametric test was chosen for its efficacy in identifying differences between two independent samples, which is essential for our study comparing two distinct graft conditions. After initial screening, the limma package (version 3.59.1) was used to refine our DEG analysis. Limma provides a robust framework for analyzing gene expression data, particularly through its ability to fit linear models for comparisons of interest and its empirical Bayes smoothing of standard errors, which enhances the reliability of DEG identification. Genes were considered differentially expressed based on two key criteria. We set the adjusted p-value threshold of less than 0.05 to ensure that the findings were statistically significant while controlling for multiple testing errors. A cutoff for a |log2-fold change| of more than 0.25 was applied. This threshold helped in identifying genes with meaningful expression differences, avoiding those with minor fluctuations that are less likely to be biologically significant. This extensive DEG analysis helped reveal the molecular variations within and across major T-cell clusters and subcell types, with detailed expression profiles visualized in various figures. We generated heatmaps to visually represent the DEGs between the syngeneic and allogeneic grafts within each T-cell subset. Heatmaps are particularly effective for this purpose because they provide a clear and intuitive visualization of the expression levels across multiple genes and conditions, facilitating quick identification of patterns and outliers in the data. This methodology not only ensures a robust analysis of gene expression differences but also helps in understanding the functional implications of these differences in the context of T-cell behavior and the immune response in islet transplantation.

### 2.3 Pathway enrichment analysis in T cell subsets

To elucidate the functional implications of differentially expressed genes (DEGs) identified within T-cell subsets from syngeneic and allogeneic islet grafts, we conducted a comprehensive pathway enrichment analysis using the HALLMARK gene set collection from the Molecular Signatures Database (MSigDB). This analysis aimed to identify key biological processes and signaling pathways that are differentially activated or suppressed across these cell subsets.

For each T-cell subset, we performed gene set enrichment analysis (GSEA) using the preranked list of genes based on their log2-fold changes. Specifically, we used the irGSEA software (version 2.1.5) and MSigDB’s mh.all.v2023.2.Mm.symbols.gmt as the gene set database. This analysis helps in identifying whether HALLMARK pathways show differences between syngeneic and allogeneic islet grafts. Pathway enrichment analysis provided detailed insights into the molecular mechanisms underlying T-cell responses in syngeneic versus allogeneic islet grafts. This comprehensive pathway enrichment analysis not only delineated the specific pathways activated or repressed in different T-cell subsets but also provided a molecular framework for understanding the potential impacts of these pathways on the fate of islet grafts.

### 2.4 Analysis of cell communication patterns in T-cell populations using CellChat

We then applied CellChat (version 2.0.0) to analyze intercellular communication by quantifying and visualizing the contributions of different ligands and receptors expressed by these cells (19). This allowed us to delineate the cellular hierarchies and communication dynamics within the T-cell population. We utilized CellChat to detect and interpret complex communication patterns among the T-cell subgroups. This included the identification of four incoming signaling patterns. These patterns represent the pathways through which T cells receive signals from other cells, helping us understand how external signals influence cell behavior and function. Three outgoing signaling patterns: These patterns illustrate how T cells send signals to other cells, indicating their role in modulating immune responses and cellular environments. This algorithm enabled the distinction between autocrine (self-signaling within the same cell type) and paracrine (signaling between different cell types) communication modes. The analysis provided a structured understanding of which cell types are the predominant senders and receivers of signals, which is critical for identifying key regulatory nodes within the immune system.

To effectively communicate our findings, we used CellChat’s built-in visualization functions. Network plots: Network plots showing the overall signaling network and highlighting the most influential cell types and pathways. Sankey diagrams: These diagrams depict the flow of signals between different cell groups, providing a clear representation of communication from senders to receivers. Heatmaps and chord diagrams: These visualizations quantified and compared the strength and frequency of interactions across different signaling pathways, emphasizing the contributions of each cell type to the overall communication network. We integrated the results from CellChat in our study to compare the signaling activities between syngeneic and allogeneic grafts. This integrative approach helped in pinpointing differential signaling pathways that might be responsible for the distinct immune responses observed between the two graft types. By employing this comprehensive methodology, we were able to uncover nuanced insights into the cellular communication landscape, revealing how specific signaling pathways are orchestrated within T-cell populations and their impact on graft outcomes.

### 2.5 Statistical analysis

All analyses were performed in R (version 4.2.1). We established statistical significance at *P* < 0.05.

## 3 Results

### 3.1 Workflow of this study

**Figure 1:**
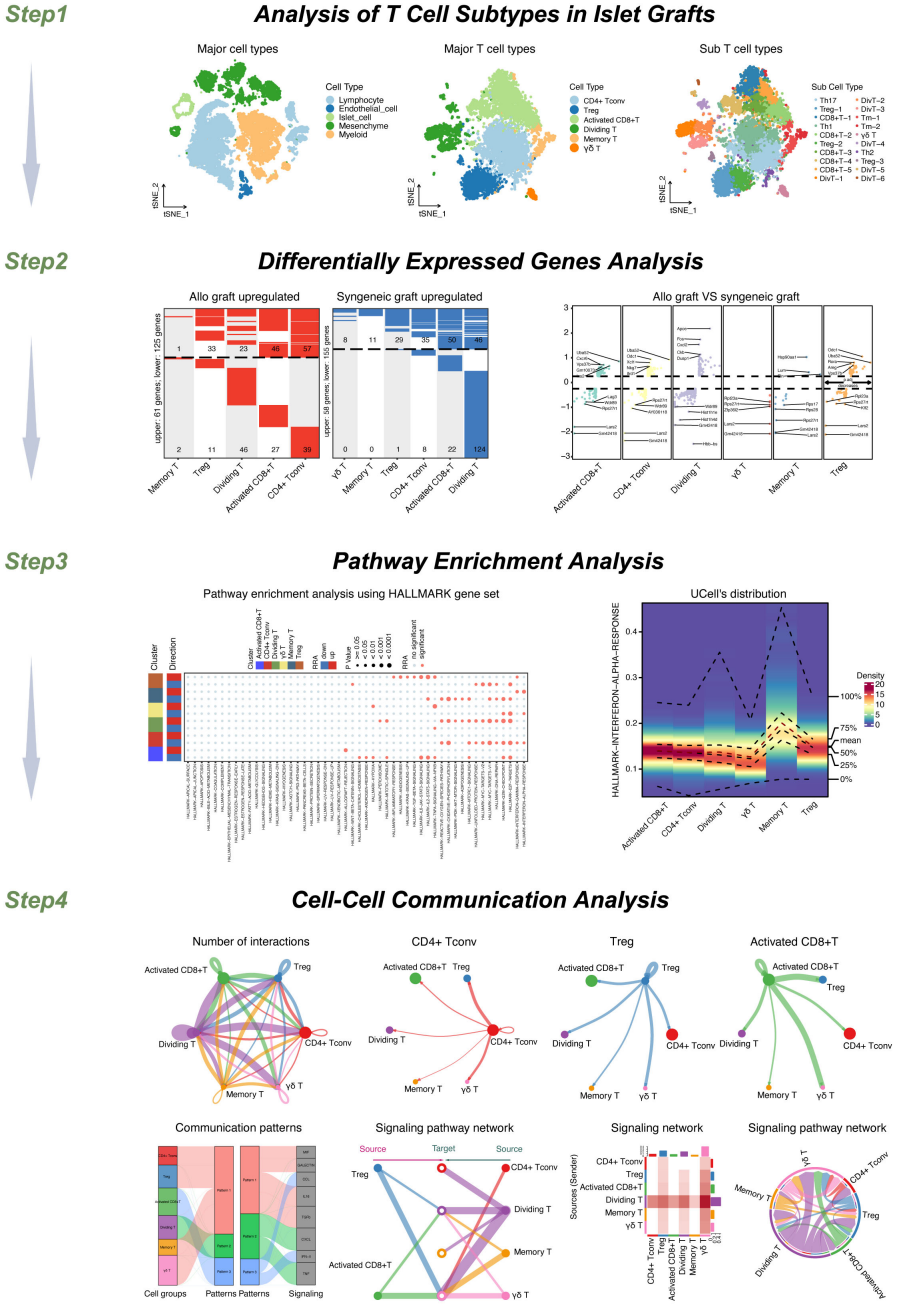
Study workflow diagram.

The workflow of this study is shown in **Figure 1**. Step 1: Our study’s workflow begins with the analysis of single-cell datasets containing both syngeneic and allogeneic islet grafts. Initial quality checks, data standardization, and preliminary dimensionality reduction set the foundation for deeper analysis using T-SNE to identify distinct cellular clusters within the grafts. Further subdivision revealed five major cell types, which were expanded into ten subtypes, enabling detailed cellular profiling and differential gene expression analysis. In-depth scRNA-seq analysis of T-cell populations revealed six transcriptionally distinct clusters. We further expanded this classification into 20 distinct subcell types.

Step 2: We then conducted a comprehensive analysis of differentially expressed genes (DEGs) across these cell types and subtypes to explore molecular differences.

Step 3: We performed pathway enrichment analyses to identify signaling pathways such as HALLMARK INTERFERON ALPHA, GAMMA RESPONSES, and TNFA SIGNALING VIA NFKB. These pathways were particularly notable for their varied expression across memory T cells, Tregs, and other T-cell types, highlighting both autocrine and paracrine signaling.

Step 4: Cell−cell interaction analyses further revealed significant ligand−receptor interactions between various T-cell subsets, emphasizing the communication dynamics within the T-cell populations. Pattern recognition techniques map these interactions, distinguishing cells as signal senders or receivers through specified signaling patterns, thus providing a comprehensive view of the communication and signaling mechanisms at play within T-cell populations. This multifaceted approach not only clarified the internal communication patterns among T cells but also linked these patterns to broader immune responses in islet transplants. Our study included a detailed analysis of the MIF signaling pathway, focusing on its impact across various T-cell subgroups. We explored autocrine signaling within the Treg and activated CD8+ T-cell groups and compared it to the paracrine signaling observed in the CD4+ Tconv, dividing T, memory T, and γδT cell groups. This process helped us assess the number of cells involved and the likelihood of communication within each subgroup.

### 3.2 Analysis of single-cell datasets

In the initial phase of our research, we obtained single-cell datasets that included syngeneic and allogeneic islet grafts from our previous study (GSE198865) (16). We conducted thorough quality checks, standardized the data, and performed initial steps to reduce dimensionality. Subsequently, we applied the T-SNE technique to achieve further dimension reduction, which allowed us to clearly distinguish cellular clusters specific to syngeneic versus allogeneic islet grafts.

**Figure 2:**
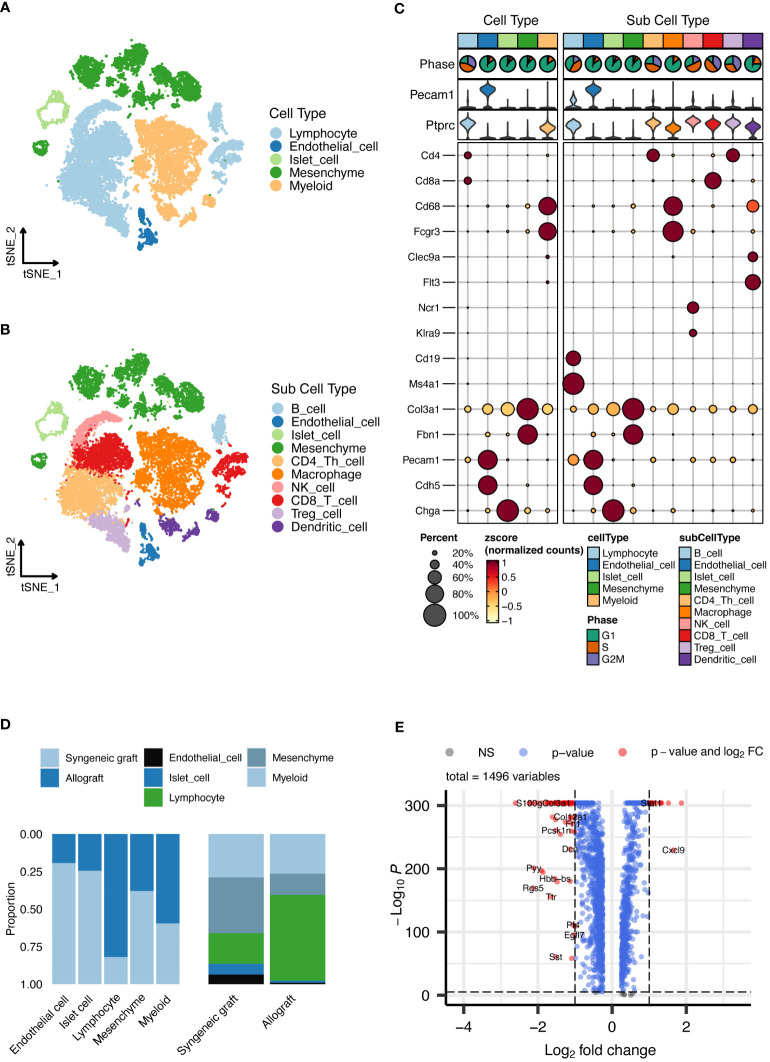
Single-cell RNA sequencing analysis of islet grafts. **(A)** Cellular clusters identified in islet grafts display the five main cell types identified within the islet grafts, which are lymphocytes, endothelial cells, islet cells, mesenchymal cells, and myeloid cells. **(B)** Subdivision of cell types showing the ten detailed subtypes of the main cell types for further cellular profiling and analysis. **(C)** Marker expression profiles showing the expression markers for each of the ten cell subtypes, providing insight into the cellular identity and function within the grafts. **(D)** The distribution of cell types documents the distribution of each cell type across the sampled grafts, highlighting variations between syngeneic and allogeneic samples. **(E)** Volcano plot of differential gene expression showing the results of the differential gene expression analysis, identifying significantly upregulated and downregulated genes.

Our analysis identified five main cell types, depicted in **Figure 2A**, which included lymphocytes, endothelial cells, islet cells, mesenchymal cells, and myeloid cells. We extended our analysis to categorize these cells into 10 detailed subtypes, as shown in **Figure 2B**, and their markers are displayed in **Figure 2C**. These subtypes consist of B cells, endothelial cells, islet cells, mesenchymal cells, CD4+ T cells, macrophages, CD8+ T cells, regulatory T cells (Tregs), natural killer (NK) cells, and dendritic cells (DCs), providing a detailed view of the cell variety within the grafts. The distribution of each cell type across the samples is thoroughly documented in **Figure 2D**. Additionally, we performed a differential gene expression analysis, the results of which are shown in a volcano plot in **Figure 2E**.

**Figure 3:**
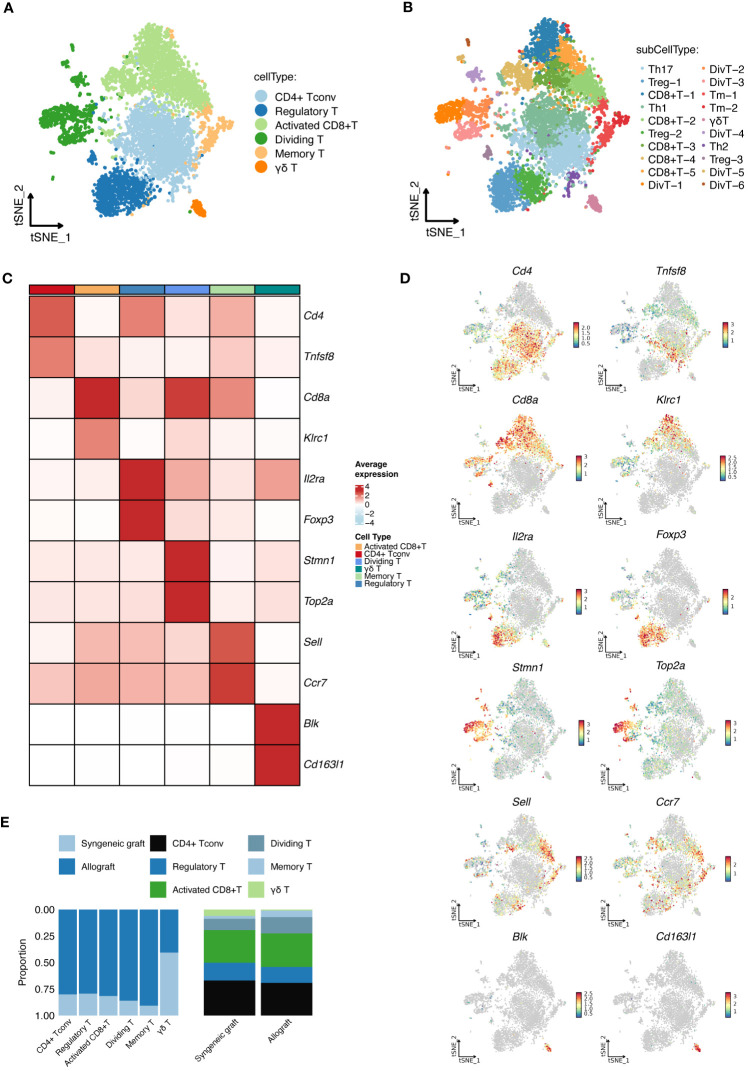
Detailed scRNA-seq analysis of T-Cell populations. **(A)** Cellular clusters of T cells identified in islet grafts show the six main cell types identified within the islet grafts. **(B)** Subdivision of T-cell types showing the 20 detailed subtypes of the main T-cell types for further cellular profiling and analysis. **(C)** The heatmap shows the specific marker profiles of different T-cell types. **(D)** The t-SNE analysis shows the specific marker profiles of different T-cell types. **(E)** The proportions of different T-cell types distributed across samples. DivT, Dividing T; Tm, Memory T.

In-depth scRNA-seq analysis of T-cell populations revealed six transcriptionally distinct clusters: CD4+ Tconv cells, Tregs, activated CD8+ T cells, dividing T cells, memory T cells, and γδ T cells, as shown in **Figure 3A**. We expanded this classification into 20 distinct subcell types, as depicted in **Figure 3B**, with the markers shown in **Figures 3C, D**. These subtypes, which include various forms of activated CD8+ T cells, dividing T cells, memory T cells, regulatory T cells, and different T helper cell types, provide a detailed perspective on the cellular diversity within grafts. The proportions of these subcell types across the samples are detailed in **Figure 3E**.

### 3.3 Comparative analysis of DEGs in T cells between syngeneic and allogeneic islet transplants

**Figure 4:**
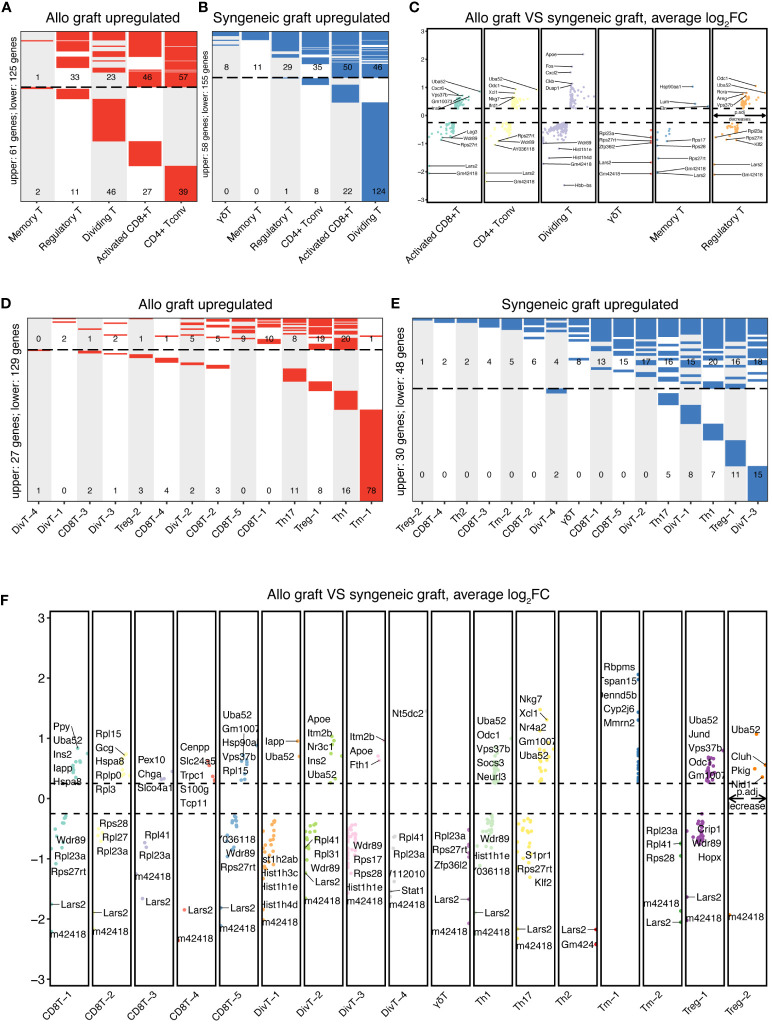
Comparative DEG analysis in T cells. The differentially expressed genes in T-cell clusters and subtypes between syngeneic and allogeneic transplants were visualized using heatmaps. **(A)** Upregulated DEGs in allogeneic T-cell clusters are upregulated within five primary T-cell clusters from allogeneic islet grafts, highlighting genes with significant expression changes. No upregulated genes were identified in γδ T cells. **(B)** Upregulated DEGs in syngeneic T-cell clusters illustrate upregulated DEGs within six primary T-cell clusters from syngeneic islet grafts, emphasizing genes with notable increases in expression. **(C)** Comparative DEG expression in T-cell clusters. Detailed comparisons of DEG expression within six primary T-cell clusters from both syngeneic and allogeneic islet grafts are presented, providing a direct visual contrast of molecular differences. **(D)** Upregulated DEGs in allogeneic T-cell subtypes across 17 subcell types of T cells in allogeneic islet grafts, delineating the specific genes that are predominantly expressed. **(E)** Upregulated DEGs in syngeneic T-cell subtypes displayed upregulated DEGs across 17 subcell types of T cells in syngeneic islet grafts, revealing genes with increased expression. **(F)** Comparative DEG expression in T-cell subtypes revealed by a detailed visual comparison of DEG expression across 17 subcell types of T cells from syngeneic versus allogeneic islet grafts, highlighting molecular distinctions.

To investigate the molecular differences between T cells from syngeneic versus allogeneic islet grafts, we conducted a thorough differential gene expression analysis. This approach enabled us to identify and characterize the DEGs across six major distinct clusters of T cells (**Figures 4A–C**) and 17 subcell types (**Figures 4D–F**). Statistical significance was established using the Wilcoxon rank-sum test and refined through the limma package, with genes considered significantly differentially expressed at an adjusted p-value < 0.05 and a |log2 fold change| > 0.25. By employing bioinformatics tools, we generated heatmaps to visually represent the DEGs between the syngeneic and allogeneic islet grafts within each T-cell subset. The upregulated DEGs within six primary T-cell clusters in allogeneic islet grafts are presented in **Figure 4A**. The upregulated DEGs within six primary T-cell clusters in syngeneic islet grafts are presented in **Figure 4B**. The detailed expression of DEGs within six primary T cells from syngeneic versus allogeneic islet grafts is presented in **Figure 4C**. The upregulated DEGs within 17 subcell types of T cells in allogeneic islet grafts are presented in **Figure 4D**. The upregulated DEGs within 17 subcell types of T cells in syngeneic islet grafts are presented in **Figure 4E**. The detailed expression of DEGs within 17 subcell types of T cells from syngeneic versus allogeneic islet grafts is presented in **Figure 4F**.

### 3.4 Pathway enrichment in T cell subsets

**Figure 5:**
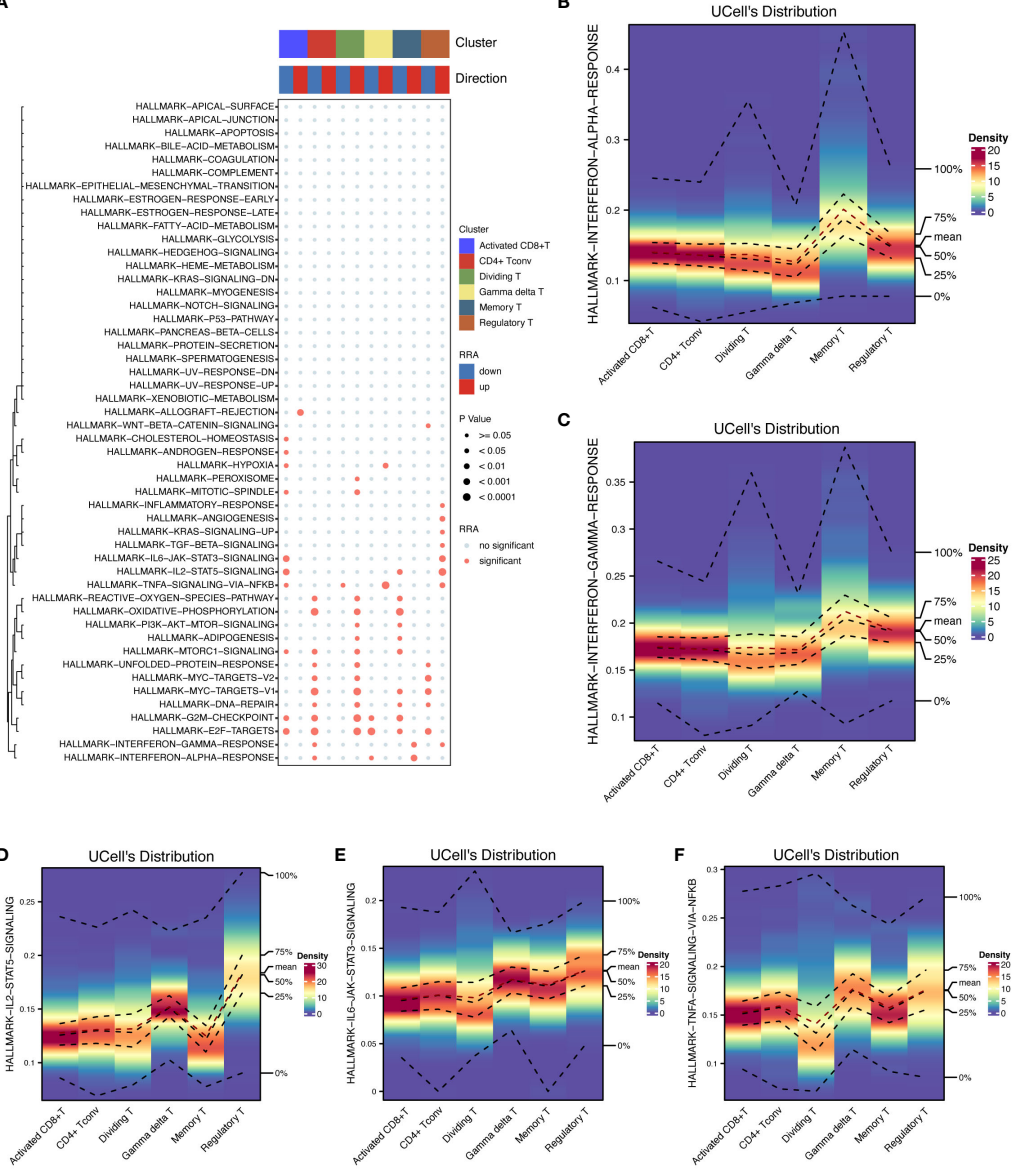
Pathway enrichment analysis. The activation of key HALLMARK signaling pathways in T cells was shown to provide insight into their roles in immune responses. **(A)** Pathway enrichment analysis details the results of pathway enrichment analysis across T-cell subsets, highlighting pathways that are differentially activated or suppressed. **(B)** HALLMARK INTERFERON ALPHA response in memory T cells quantifies the INTERFERON ALPHA response activation in memory T cells. **(C)** HALLMARK INTERFERON GAMMA Response in T cells shows an elevated INTERFERON GAMMA response in memory T cells and Tregs and reduced levels in CD4+ Tconv cells. **(D)** HALLMARK IL2-STAT5 signaling in Treg cells emphasizes strong IL2-STAT5 pathway activation in Treg cells. **(E)** HALLMARK IL6-STAT3 signaling in Treg cells is highly active in the IL6-STAT3 signaling pathway in Treg cells. **(F)** HALLMARK TNFA SIGNALING VIA NFKB in Treg cells illustrates the activation of TNFA SIGNALING VIA NFKB in Tregs, γδT cells, dividing T cells, and activated CD8+ T cells.

Furthermore, we conducted pathway enrichment analysis on these cellular subsets (**Figure 5A**). This analysis highlighted that the HALLMARK INTERFERON ALPHA RESPONSE pathway was predominantly activated in memory T cells, whereas it was suppressed in CD4+ Tconv cells and γδT cells. Similarly, the HALLMARK INTERFERON GAMMA RESPONSE was elevated in memory T cells and Tregs but reduced in CD4+ Tconv cells. Additionally, the HALLMARK TNFA SIGNALING VIA NFKB pathway was prominently active in Tregs, γδT cells, dividing T cells, and activated CD8+ T cells. The HALLMARK IL2 STAT5 SIGNALING pathway exhibited increased activity in Tregs but decreased activity in both memory T cells and activated CD8+ T cells.

In terms of the expression levels and distribution of differentially expressed genes among the cell subsets, the highest intensity of genes in the memory T cells was detected in the HALLMARK INTERFERON ALPHA (**Figure 5B**) and GAMMA RESPONSES (**Figure 5C**) subsets, each of which was marked at approximately 0.2 in the respective figures. Treg cells showed the most significant changes in the expression of HALLMARK IL2-STAT5 (**Figure 5D**), IL6-STAT3 SIGNALING (**Figure 5E**), and HALLMARK TNFA SIGNALING VIA NFKB (**Figure 5F**), with intensities of approximately 0.15, 0.1, and 0.15, respectively.

### 3.5 Analysis of cell−cell interactions in T cells

**Figure 6:**
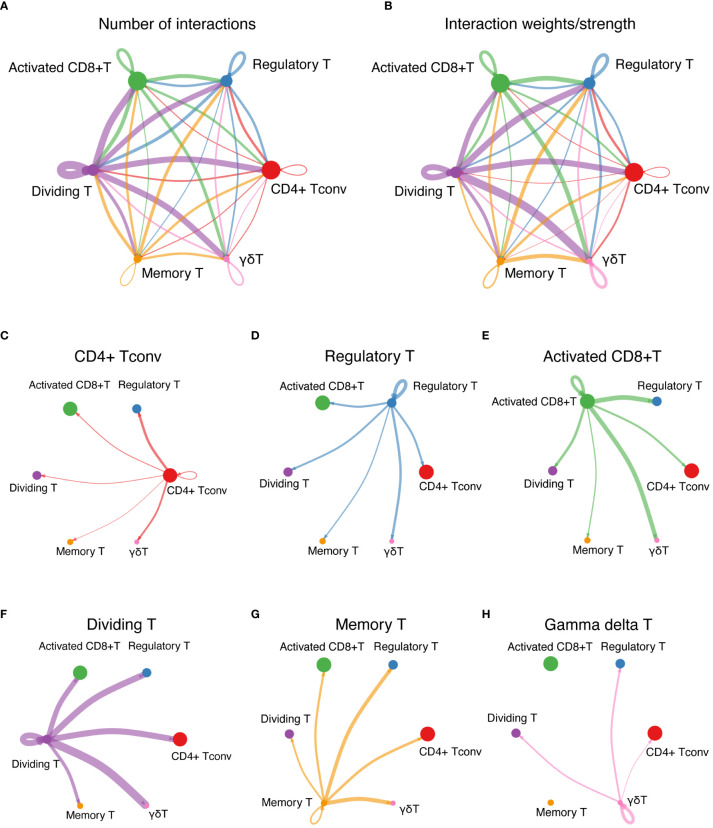
Cell-cell interaction network. **(A)** The interactions among T cells represent the number of ligand-receptor interactions among various T-cell types, emphasizing network complexity. **(B)** Interaction strength among T cells shows the interaction weights or strengths among various T-cell types, providing insights into the intensity of cellular communication. **(C-H)** Detailed ligand−receptor interactions. **(C)** through **(H)** show the strong interactions between specific pairs of T-cell types, revealing the key pathways and mediators involved in cellular communication.

Pronounced ligand−receptor interactions were observed across various T-cell types, with notable exchanges between dividing T cells and activated CD8+ T cells, Tregs, CD4+ Tconv cells, and γδT cells (**Figures 6A, B**). The number of interactions among these cells is shown in **Figure 6A**, and the interaction weights/strengths are shown in **Figure 6B**. **Figures 6C–H** shows the details of the ligand−receptor interactions between various T-cell types. **Figure 6C** shows strong interactions between CD4+ Tconvs and Tregs and between CD4+ Tconvs and γδT cells. **Figure 6D** shows the strong interactions between Tregs and γδT cells. **Figure 6E** shows the strong interactions between activated CD8+ T cells and both γδT cells and Tregs. **Figure 6F** shows strong interactions between dividing T cells and γδT cells, Tregs, CD4+ Tconvs and activated CD8+ T cells. **Figure 6G** shows strong interactions between memory T cells and Tregs and between memory T cells and γδT cells. **Figure 6H** shows the strong interactions between γδT cells and Tregs and between γδT cells and dividing T cells.

### 3.6 Analyzing cell communication patterns and signaling pathways in T-cell populations

**Figure 7:**
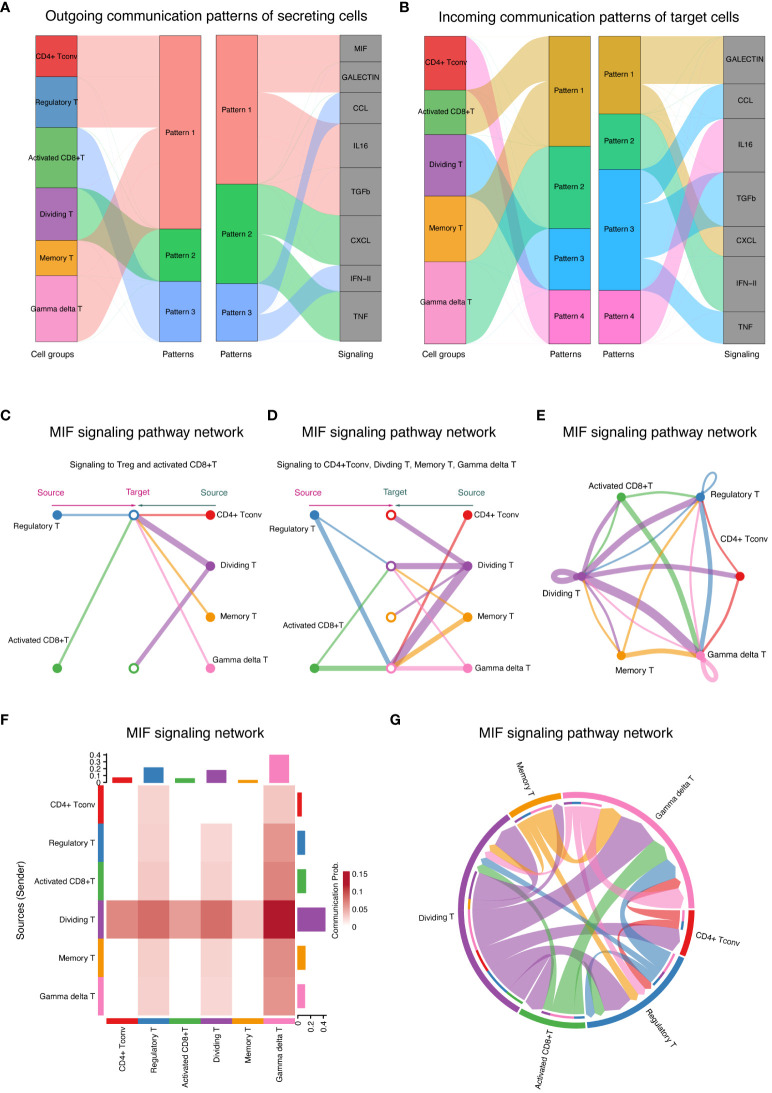
Cell communication patterns and signaling pathways **(A)** Signaling patterns for signal receivers showing signaling patterns for T-cell groups acting as signal receivers, detailing the pathways stimulated by ligands. **(B)** Signaling Patterns for Signal Senders Illustrate signaling patterns for T-cell groups acting as signal senders, highlighting the pathways through which these cells secrete ligands. **(C)** MIF signaling pathway autocrine and paracrine signaling in T cells demonstrates the impact of the MIF signaling pathway on Tregs and activated CD8+ T cells, showing autocrine (left) and paracrine (right) signaling. **(D)** Signaling modulation among T-cell subtypes depicts autocrine and paracrine signaling within CD4+ Tconv cells, dividing T cells, memory T cells, and γδT cells. The circle sizes and line widths represent the number of cells and the potential strength of communication, respectively. **(E)** Ring chart visualization of the distribution of the MIF signaling pathway in the T-cell group. A ring chart showing the distribution and role prominence of various T-cell groups within the MIF signaling pathway. **(F)** The heatmap of role prominence in MIF signaling displays a heatmap showing the likelihood of role assumption by different T-cell groups, with color intensity indicating each group’s role prominence. **(G)** Chord diagram of T-cell communication in MIF signaling. A chord diagram maps the network of T-cell communications within the MIF signaling pathway, with chord thickness reflecting the strength and frequency of interactions, particularly highlighting the role of γδT cells as central mediators.

The patterns of cell communication for groups that primarily acted as signal receivers (cells stimulated by ligands) are shown in **Figure 7A**. The width of the flow in the diagram indicates the contribution of each element to the pattern. CD4+ Tconv, Treg, memory T, and γδT cells mainly receive stimuli through pattern #1, which includes the MIF, GALECTIN, IL16, and TGFb signaling pathways. Activated CD8+ T cells predominantly receive stimuli through pattern #3, which consists solely of the CCL and IFN-II signaling pathways. Dividing T cells primarily receive stimuli through pattern #2, which includes only the CXCL and TNF signaling pathways.

The pattern of cell communication in groups primarily serving as signal senders (secretion ligands) is shown in **Figure 7B**. Most of the CD4+ Tconv cells that secrete ligands communicate via pattern #4, which includes the IL16 signaling pathway. All activated CD8+ T cells and memory T cells communicate via pattern #1, which encompasses the GALECTIN and CXCL signaling pathways. All dividing T cells communicate via pattern #3, which includes the CCL, TGFb, and TNF signaling pathways. All γδT cells communicate via pattern #2, which includes the IFN-II signaling pathway.

By comparing **Figures 7A, B**, we can distinguish between autocrine and paracrine links. Specifically, the MIF and TGFb signaling pathways primarily mediate autocrine signaling, while the CCL and CXCL pathways mainly facilitate paracrine communication between activated CD8+ T cells and dividing T cells.

### 3.7 Analyzing autocrine and paracrine signaling in T cells via the MIF signaling pathway

The MIF signaling pathway was further analyzed, revealing its impact on various T-cell subgroups (**Figure 7C**). On the left, the diagram illustrates autocrine signaling within the Treg and activated CD8+ T-cell groups, while the right side shows their paracrine signaling. **Figure 7D** divides the focus into two types of signaling affecting CD4+ Tconv cells: dividing T cells, memory T cells, and γδT cells. The left side of the diagram represents paracrine signaling in these groups, and the right side illustrates autocrine signaling in the same groups. The size of the circles in the diagram indicates the number of cells in each group, and the width of the lines suggests the likelihood of cell communication occurring.

Detailed visualizations that complement the insights into the MIF signaling pathway are provided in **Figures 7E–G**. A ring chart illustrates the distribution and prominence of various T-cell groups within the MIF signaling pathway, highlighting their respective contributions and interactions (**Figure 7E**). The positioning and size of each segment within the ring chart correlate with the role prominence of each cell group, as derived from the heatmap (**Figure 7F**). A chord diagram was further used to map the intricate network of cell communication within the MIF signaling pathway (**Figure 7G**). This diagram shows the connections between different T-cell groups, with the thickness of each chord representing the strength and frequency of interactions between the groups. As in **Figure 7F**, where the heatmap depicts the likelihood of role assumption, the chord diagram in **Figure 7G** visually emphasizes the central role of γδT cells, as they act as the main mediators managing signal flow during cell communication. Together, these results underscore the dynamics of cellular interactions, and the crucial roles certain T-cell groups play within the signaling pathway.

## 4 Discussion

Islet transplantation has emerged as a promising therapeutic strategy for patients suffering from type 1 diabetes (20, 21). Allogeneic islet transplantation offers potential cures, yet they are hindered by immune rejection and the scarcity of compatible donors (8). This study aimed to address these challenges by dissecting the molecular mechanisms underlying T-cell responses in both transplantation scenarios, highlighting the necessity for a deeper understanding of immune dynamics to improve transplant outcomes.

Immune rejection in allogeneic islet transplantation is directly linked to the loss of functional pancreatic islets (22, 23). By elucidating the T-cell-mediated immune responses that contribute to islet graft rejection, this research endeavors to unlock new avenues for enhancing graft survival and function (24). The insights gained from our investigation into the differential gene expression and signaling pathways of T-cell subsets in allogeneic and syngeneic transplants could shed light on novel immunomodulatory therapies, potentially revolutionizing the management of type 1 diabetes and improving patient prognoses.

Given the complexity of immune responses in islet transplantation, identifying differentially expressed genes (DEGs) in T-cell clusters is crucial. The upregulation of specific genes within the major T-cell clusters and subtypes in syngeneic transplants suggests a unique molecular signature potentially linked to graft rejection or other immune responses. These DEGs could serve as biomarkers for transplant outcomes or therapeutic targets to enhance graft survival.

Our single-cell transcriptomic analysis has provided significant insights into T-cell heterogeneity and its molecular mechanisms in islet transplantation. Notably, the enrichment of the HALLMARK INTERFERON ALPHA RESPONSE pathway in memory T cells indicates a heightened antiviral defense, which is crucial for graft survival. Conversely, the suppression of this pathway in CD4+ helper and γδ T cells likely represents a regulatory mechanism to prevent tissue damage. Additionally, the widespread activation of the HALLMARK TNFA SIGNALING VIA NFKB pathway across various T-cell subsets, including regulatory T (Treg) cells, γδ T cells, proliferating T cells, and activated CD8+ T cells, highlights the critical role of TNFα in mediating inflammatory responses, which may be targeted to modulate graft rejection and enhance tolerance. The activation of the HALLMARK TNFA SIGNALING VIA NFKB across multiple T-cell subsets suggests a heightened inflammatory state which could predispose to graft rejection or dysfunction. Conversely, the suppression of the INTERFERON ALPHA RESPONSE in CD4+ Tconv and γδT cells may represent a compensatory, regulatory mechanism aimed at tempering the immune response to avoid overactivation and potential graft damage.

The interaction between proliferating T cells and activated CD8+ T cells, as revealed through ligand−receptor analysis using CellChat, underscores the complexity of communication within the T-cell community. These interactions are pivotal for orchestrating the immune response to transplanted tissues, highlighting their potential as targets for enhancing graft acceptance and preventing rejection. Similar challenges are observed in other types of organ transplantation, where post-transplantation diabetes mellitus (PTDM) emerges as a serious complication affecting graft and patient survival (25).

Furthermore, the analysis revealed the critical role of the MIF signaling pathway in modulating interactions among various T-cell subtypes. This pathway is influenced by autocrine signaling in Tregs and activated CD8+ T cells, as well as paracrine signaling in CD4+ conventional T (CD4+ Tconv), proliferating T, memory T, and γδ T cells. This complex signaling pathway highlights the integral role of MIF in immune regulation and suggests that detailed insights into this pathway could inform strategies to modulate immune responses in transplant settings. Moreover, the significant role of MIF in various biological processes and immune responses, particularly its impact on different T-cell types, underscores its potential as a biomarker or therapeutic target in islet transplantation. Compared to findings from the previous study (11), our study further elaborates on the role of γδ T cells in graft environments, providing a deeper understanding of their dual role in immunoregulation and inflammation.

Our findings highlight the potential of targeting specific T-cell signaling pathways, such as TNFA via NFKB, to modulate the immune response in islet transplantation. These pathways hold promise as therapeutic targets to enhance graft tolerance. However, the limited sample size in our study may affect the generalizability of these results. To address this, further studies should investigate the role of these pathways in larger cohorts to explore the mechanistic basis of their modulation using targeted therapies or genetic techniques. Additionally, we recommend conducting future clinical trials designed to assess interventions aimed at the TNFA and interferon pathways, which have been identified as critical in our study. Such trials could provide deeper insights into their potential to improve transplantation outcomes and validate our findings across a broader population, thereby enhancing their applicability and impact in clinical settings.

In summary, our study highlights the intricate interplay of T-cell subsets and their communication networks, which are crucial for understanding immune responses in pancreatic islet transplants. Through detailed analyses using GSEA and CellChat, we identified specific biological processes and signaling pathways that are differentially regulated across T-cell subpopulations. These insights not only deepen our understanding of T-cell behavior in the context of transplantation but also offer potential avenues for developing targeted immunomodulatory therapies aimed at improving transplant tolerance and longevity.

## Data availability statement

The original contributions presented in the study are included in the article/supplementary material. Further inquiries can be directed to the corresponding authors.

## Author contributions

HZ: Writing – original draft, Writing – review & editing. ZP: Conceptualization, Writing – review & editing, Funding acquisition. YL: Formal analysis, Writing – review & editing. PZ: Funding acquisition, Writing – review & editing. HY: Project administration, Writing – review & editing, Funding acquisition. LM: Conceptualization, Funding acquisition, Project administration, Writing – original draft, Writing – review & editing.

## References

[1] Subramanian S Khan F Hirsch IB. New advances in type 1 diabetes. BMJ. (2024) 384:e075681. doi: 10.1136/bmj-2023-075681 38278529

[2] Quattrin T Mastrandrea LD Walker LSK. Type 1 diabetes. Lancet. (2023) 401:2149–62. doi: 10.1016/S0140-6736(23)00223-4 37030316

[3] Martens P-J Mathieu C. Type 1 diabetes mellitus: A brave new world. Nat Rev Endocrinol. (2024) 20:71–2. doi: 10.1038/s41574-023-00936-y 38057482

[4] Kaur J Seaquist ER. Hypoglycaemia in type 1 diabetes mellitus: Risks and practical prevention strategies. Nat Rev Endocrinol. (2023) 19:177–86. doi: 10.1038/s41574-022-00762-8 36316392

[5] Herold KC Delong T Perdigoto AL Biru N Brusko TM Walker LSK. The immunology of type 1 diabetes. Nat Rev Immunol. (2024) 24:435–51. doi: 10.1038/s41577-023-00985-4 PMC7616056 38308004

[6] Wu J Li T Guo M Ji J Meng X Fu T. Treating a type 2 diabetic patient with impaired pancreatic islet function by personalized endoderm stem cell-derived islet tissue. Cell Discovery. (2024) 10:45. doi: 10.1038/s41421-024-00662-3 38684699 PMC11058776

[7] Kioulaphides S García AJ. Encapsulation and immune protection for type 1 diabetes cell therapy. Adv Drug Delivery Rev. (2024) 207:115205. doi: 10.1016/j.addr.2024.115205 PMC10948298 38360355

[8] Lansberry TR Stabler CL. Immunoprotection of cellular transplants for autoimmune type 1 diabetes through local drug delivery. Adv Drug Delivery Rev. (2024) 206:115179. doi: 10.1016/j.addr.2024.115179 PMC11140763 38286164

[9] Mullard A. FDA approves first cell therapy for type 1 diabetes. Nat Rev Drug Discovery. (2023) 22:611. doi: 10.1038/d41573-023-00113-w 37419946

[10] Cayabyab F Nih LR Yoshihara E. Advances in pancreatic islet transplantation sites for the treatment of diabetes. Front Endocrinol (Lausanne). (2021) 12:732431. doi: 10.3389/fendo.2021.732431 34589059 PMC8473744

[11] Short S Lewik G Issa F. An immune atlas of T cells in transplant rejection: pathways and therapeutic opportunities. Transplantation. (2023) 107:2341–52. doi: 10.1097/TP.0000000000004572 PMC10593150 37026708

[12] Wang J Zhao X Wan YY. Intricacies of TGF-β signaling in Treg and Th17 cell biology. Cell Mol Immunol. (2023) 20:1002–22. doi: 10.1038/s41423-023-01036-7 PMC10468540 37217798

[13] Bernaldo-de-Quirós E López-Abente J Camino M Gil N Panadero E López-Esteban R. The presence of a marked imbalance between regulatory T cells and effector T cells reveals that tolerance mechanisms could be compromised in heart transplant children. Transplant Direct. (2021) 7:e693. doi: 10.1097/TXD.0000000000001152 33928185 PMC8078462

[14] Wen L Li G Huang T Geng W Pei H Yang J. Single-cell technologies: From research to application. Innovation (Camb). (2022) 3:100342. doi: 10.1016/j.xinn.2022.100342 36353677 PMC9637996

[15] Hao Y Hao S Andersen-Nissen E Mauck WM Zheng S Butler A. Integrated analysis of multimodal single-cell data. Cell. (2021) 184:3573–3587.e29. doi: 10.1016/j.cell.2021.04.048 34062119 PMC8238499

[16] Chen P Yao F Lu Y Peng Y Zhu S Deng J. Single-cell landscape of mouse islet allograft and syngeneic graft. Front Immunol. (2022) 13:853349. doi: 10.3389/fimmu.2022.853349 35757709 PMC9226584

[17] Korsunsky I Millard N Fan J Slowikowski K Zhang F Wei K. Fast, sensitive and accurate integration of single-cell data with Harmony. Nat Methods. (2019) 16:1289–96. doi: 10.1038/s41592-019-0619-0 PMC6884693 31740819

[18] Laurens van der M Hinton G. Visualizing Data using t-SNE. Mach Learn Res. (2008) 9:2579–605.

[19] Jin S Guerrero-Juarez CF Zhang L Chang I Ramos R Kuan C-H. Inference and analysis of cell-cell communication using CellChat. Nat Commun. (2021) 12:1088. doi: 10.1038/s41467-021-21246-9 33597522 PMC7889871

[20] Chetboun M Drumez E Ballou C Maanaoui M Payne E Barton F. Association between primary graft function and 5-year outcomes of islet allogeneic transplantation in type 1 diabetes: A retrospective, multicentre, observational cohort study in 1210 patients from the Collaborative Islet Transplant Registry. Lancet Diabetes Endocrinol. (2023) 11:391–401. doi: 10.1016/S2213-8587(23)00082-7 37105208 PMC10388704

[21] Bornstein SR Ludwig B Steenblock C. Progress in islet transplantation is more important than ever. Nat Rev Endocrinol. (2022) 18:389–90. doi: 10.1038/s41574-022-00689-0 PMC9109192 35578026

[22] Chen Q-D Liu L Zhao X-H Liang J-B Li S-W. Challenges and opportunities in the islet transplantation microenvironment: a comprehensive summary of inflammatory cytokine, immune cells, and vascular endothelial cells. Front Immunol. (2023) 14:1293762. doi: 10.3389/fimmu.2023.1293762 38111575 PMC10725940

[23] Wang Q Huang Y-X Liu L Zhao X-H Sun Y Mao X. Pancreatic islet transplantation: current advances and challenges. Front Immunol. (2024) 15:1391504. doi: 10.3389/fimmu.2024.1391504 38887292 PMC11180903

[24] Kaufman DB Platt JL Rabe FL Dunn DL Bach FH Sutherland DE. Differential roles of Mac-1+ cells, and CD4+ and CD8+ T lymphocytes in primary nonfunction and classic rejection of islet allografts. J Exp Med. (1990) 172:291–302. doi: 10.1084/jem.172.1.291 2113565 PMC2188153

[25] Wu J Li T Guo M Ji J Meng X Fu T. Progress of new-onset diabetes after liver and kidney transplantation. Cell Discovery. (2023) 10:1091843. doi: 10.3389/fendo.2023.1091843 PMC9944581 36843576

